# Cisplatin modulates B-cell translocation gene 2 to attenuate cell proliferation of prostate carcinoma cells in both p53-dependent and p53-independent pathways

**DOI:** 10.1038/srep05511

**Published:** 2014-07-01

**Authors:** Kun-Chun Chiang, Ke-Hung Tsui, Li-Chuan Chung, Chun-Nan Yeh, Tsui-Hsia Feng, Wen-Tsung Chen, Phei-Lang Chang, Hou-Yu Chiang, Horng-Heng Juang

**Affiliations:** 1 Department of General Surgery, Chang Gung Memorial Hospital, Keelung, Taiwan, ROC; 2 Department of Urology, Chang Gung Memorial Hospital, Kwei-Shan, Tao-Yuan, Taiwan, ROC; 3 Department of Anatomy, College of Medicine, Chang Gung University, Kwei-Shan, Tao-Yuan, Taiwan, ROC; 4 Department of General Surgery, Chang Gung Memorial Hospital, Kwei-Shan, Tao-Yuan, Taiwan, ROC; 5 School of Nursing, College of Medicine, Chang Gung University, Kwei-Shan, Tao-Yuan, Taiwan, ROC; 6 National Kaohsiung University of Hospitality and Tourism, Hsiao-Kang, Kaohsiung Taiwan R.O.C; 7 These authors contributed equally to this work.

## Abstract

Cisplatin is a widely used anti-cancer drug. The B-cell translocation gene 2 (BTG2) is involved in the cell cycle transition regulation. We evaluated the cisplatin effects on prostate cancer cell proliferation and the expressions of BTG2, p53, androgen receptor (AR) and prostate specific antigen (PSA) in prostate carcinoma, p53 wild-type LNCaP or p53-null PC-3, cells. Cisplatin treatments attenuated cell prostate cancer cell growth through inducing Go/G1 cell cycle arrest in lower concentration and apoptosis at higher dosage. Cisplatin treatments enhanced p53 and BTG2 expression, repressed AR and PSA expression, and blocked the activation of androgen on the PSA secretion in LNCaP cells. BTG2 knockdown in LNCaP cells attenuated cisplatin-mediated growth inhibition. Cisplatin enhanced BTG2 gene expression dependent on the DNA fragment located within -173 to -82 upstream of BTG2 translation initiation site in prostate cancer cells. Mutation of the p53 response element from GGGCAGAGCCC to GGGCACC or mutation of the NFκB response element from GGAAAGTCC to GGAAAGGAA by site-directed mutagenesis abolished the stimulation of cisplatin on the BTG2 promoter activity in LNCaP or PC-3 cells, respectively. Our results indicated that cisplatin attenuates prostate cancer cell proliferation partly mediated by upregulation of BTG2 through the p53-dependent pathway or p53-independent NFκB pathway.

Prostate cancer, ranking as the second most common solid tumor for men in United States, has caused 28,170 patients dying of this disease in 2012^1^. Although with the improvement in measurement technique of detection biomarker prostate-specific antigen (PSA) for prostate cancer, leading to the early diagnosis of prostate cancer more likely, the high risk prostate cancer patients still have high recurrence rate and distant metastasis^2^,^3^. Even under multimodal approaches, 10–25% prostate cancer patients still die of metastatic disease^3^.

Cisplatin, a neutral inorganic and square planar complex, functions through binding with DNA to form adduct to induce unique specific cellular responses, mainly culminating in apoptosis induction^4^. Since the application of cisplatin in clinical trial to treat cancer, cisplatin has brought a substantial impact on cancer treatment and changed the therapeutic regimens for a number of cancers including prostate^5^,^6^. Although the clinical benefits brought by cisplatin usage is obvious, the exact mechanism of how cisplatin exerts its antitumor effect is still not very clear, although the main mechanism is recognized as activation of p53^7^.

The B-cell translocation gene 2 (BTG2), belonging to antiproliferative APRO family proteins^8^ featuring highly conservative domains, the BTG-Box A (Y^50^–N^71^) and BTG-Box B (L^97^–E^115^), is located mainly in the cytoplasm and functions in a variety of important cellular responses^9^. In terms of cancer cells, BTG2 acts as a tumor suppressor gene in a number of cancers and is activated mainly by p53 dependent pathway subsequent to DNA damage^10^,^11^. The p53 independent BTG2 expression is also possible through the PKC-δ pathway in p53-null cancer cells^12^. Regarding prostate cancer, our previous studies have indicated that ectopic overexpression of BTG2 in PC-3 cells, a p53-null prostate cancer cell line, was able to inhibit cancer cell proliferation^13^.

Our previous study has shown that topoisomerase inhibitors could repress prostate cancer cell growth and induce BTG2 expressions in a p53 dependent manner^14^. Our objectives for this study are to determine the effects of cisplatin on prostate cancer cell growth, the AR and PSA expression, as well as the regulatory mechanisms of cisplatin on the gene expression of BTG2 in prostate cancer cells.

## Results

**Figure 1:**
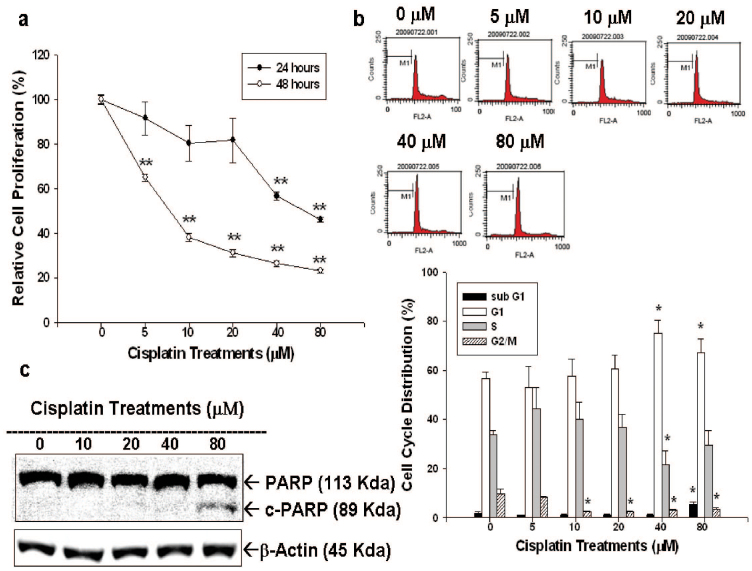
Cisplatin regulates cell proliferation and cell cycle progression in LNCaP cells. (a) LNCaP cells were treated with various concentrations of cisplatin, as indicated, for 24 (black circle) and 48 (white circle) hours and the cell proliferation was determined by the H^3^-thymidine incorporation assay. (b) LNCaP cells were serum starved for 24 hours and then were treated with 0–80 μM of cisplatin as indicated for 24 hours. The cells were stained with PI, and the cell cycle distribution was analyzed by flow cytometry. Each box represents the mean ± SE (n = 6). (c) LNCaP cells were treated with indicated concentrations of cisplatin for 24 hours. Cells were lysed and expressions of PARP, cleaved PARP (c-PARP) were investigated with β-actin serving as an internal control.(Cropped gel) (*P < 0.05, **P < 0.01).

After different concentrations of cisplatin treatment (0–80 μM) for 24 or 48 hours, cell proliferation of LNCaP cells were measured by ^3^H-thymidine incorporation assay (Fig 1a). Our results indicated LNCaP cell proliferation was inhibited by 24 hours of cisplatin treatment in a dose-dependent manner, with 41% and 50% decreases noted when treated with 40 and 80 μM cisplatin, respectively. 48 hours cisplatin treatment clearly showed more prominent cell proliferation inhibition in LNCaP cells under the concentrations from 5 to 80 μM. Results from flow cytometric analysis of LNCaP cells revealed that 40 μM of cisplatin treatment induced 15% increase in G1 phase cell together with a decrease in S phase cells in LNCaP cells after 24 hours incubation, indicating 40 μM cisplatin treatment induced G1/S arrest in LNCaP cells. Further, since 80 μM of cisplatin increased the sub-G1 fraction of cells by 7–10%, it clearly indicated high dose of cisplatin was able to induced LNCaP cell apoptosis (Figure 1b). This is also supported by the immunoblot assay revealing that treatment with 80 μM of cisplatin induced the expression of cleaved form of PARP in LNCaP cells (Figure 1c).

**Figure 2:**
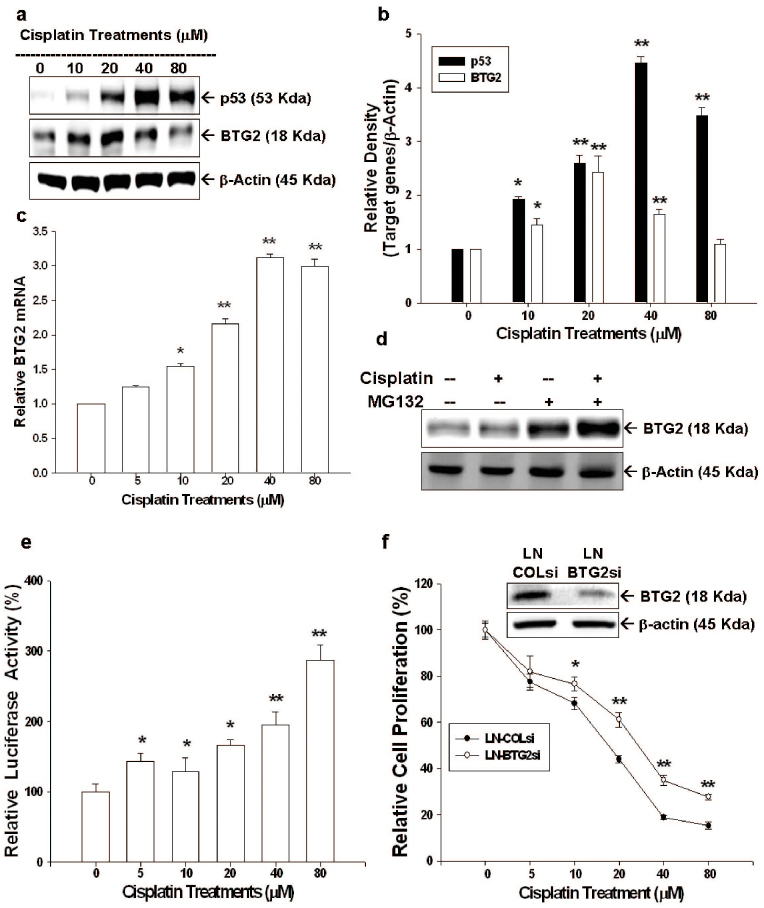
Cisplatin modulates p53 and BTG2 expression in LNCaP cells and BTG2 mediates partial cisplatin-induced growth inhibition in LNCaP cells. (a) LNCaP cells were treated with various concentrations of cisplatin, as indicated, for 24 hours. Cells were lysed and expressions of BTG2 and p53 were determined by immunoblotting assay.(Cropped gel) (b). Data of quantitative immunoblot analysis are expressed as relative density (mean ± SE; n = 3). (c) LNCaP cells were treated with various concentrations of cisplatin, as indicated, for 24 hours. BTG2 mRNA levels were determined by RT-qPCR. (d) LNCaP cells were pretreated with or without MG132 for 2 hours and then treated with 80 μM cisplatin for 24 hours. Cells were lysed and expression of BTG2 was determined by immnuoblotting assay.(Cropped gel) (e) Luciferase activity of BTG2 reporter vectors-transfected LNCaP cells after treated with various concentrations of cisplatin for 24 hours. (f) LN-BTGsi cells (BTG2 knockdown LNCaP cells) and LN-COLsi cells (mock-knockdown LNCaP cells) were treated with indicated concentrations of cisplatin for 48 hours. Cell proliferation was measured by the CyQUANT cell proliferation assay. Each value is presented as the % in relation to the control (solvent-treated) group. Data are shown as the mean percentage ± SE (n = 6) (*P < 0.05, **P < 0.01).

The results from immunoblot assay demonstrated that upregulation of BTG2 and p53 expression was noted when LNCaP cells were treated with different concentrations of cisplatin (Figure 2a). Quantitative analysis of the immunoblot results revealed a 4.5-fold and 2.4-fold increases of p53 and BTG2 protein levels were observed after 40 or 20 μM cisplatin treatments (Figure 2b). Of note, instead of increasing steadily, 80 μM cisplatin decreased the BTG2 expression as compared to 40 μM cisplatin in LNCaP cells (Figure 2b). Results of RT-qPCR indicated that cisplatin treatments induced BTG2 mRNA levels in dosage-dependent manner (Figure 2c). The reason behind this is since this concentration of cisplatin induced LNCaP cells apoptosis, which, in turn, degraded BTG2 protein via ubiquitin-proteasome system, which has been proved in our previous study^14^. Treatment of MG132, a proteasome inhibitor, increased BTG2 protein levels in LNCaP cells; moreover, when cotreatment of MG132 and 80 μM cisplatin, BTG2 expression was partially restored (Figure 2d). The results of transient gene expression assay using the reporter vector containing the promoter (−297 to −1) of human BTG2 gene indicated that cisplatin induced human BTG2 promoter activity dose-dependently (Figure 2e), in line with the previous immunoblot and RT-qPCR finding. To further verity BTG2's role in cisplatin-mediated LNCaP cell proliferative inhibition, we knocked down BTG2 in LNCaP cells (LN-BTG2si) cells by shRNA. Results of CyQUANT cell proliferation assay revealed that LN-BTG2si cells clearly showed less proliferative inhibition than LN-COLsi (mock knockdown BTG2 LNCaP cells) cells as treated by cisplatin, indicating cisplatin represses LNCaP cell growth partly mediated by downregulation of BTG2 expression (Figure 2f).

**Figure 3:**
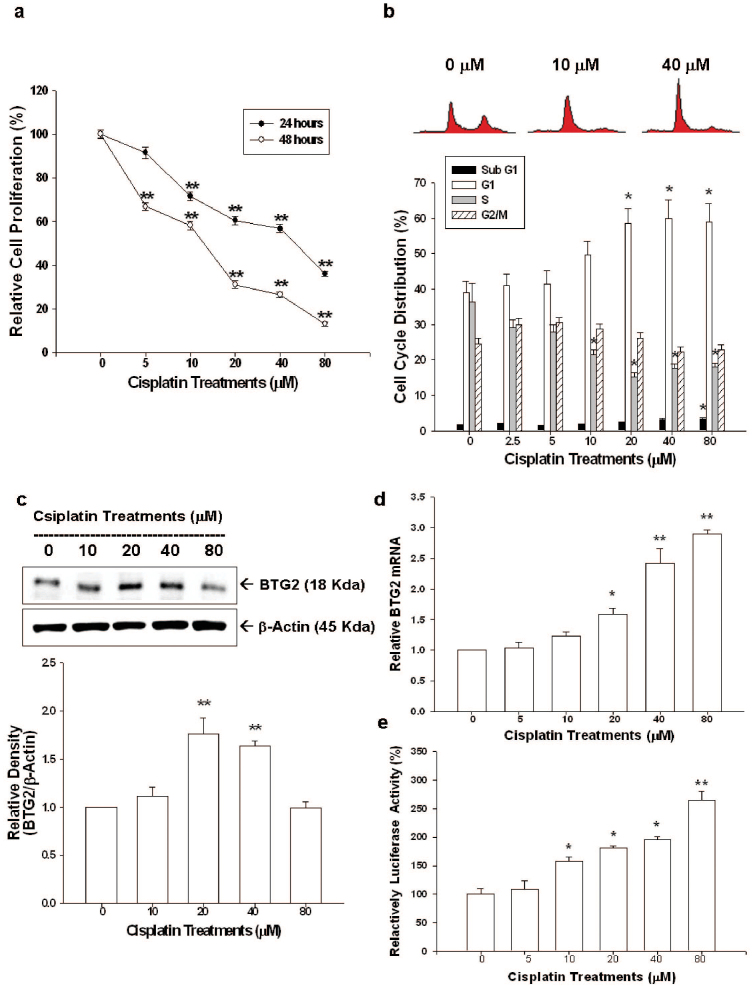
Cisplatin regulates cell proliferation, cell cycle progression and BTG2 expression in PC-3 cells. (a) PC-3 cells were treated with various concentrations of cisplatin for 24 (black circle) or 48 (white circle) hours. Cell proliferation was determined by the H^3^-thymidine incorporation assay. (b) PC-3 cells were serum starved for 24 hours and then were treated with 0–80 μM of cisplatin as indicated for 24 hours. The cells were stained with PI, and the cell cycle distribution was analyzed by flow cytometry. Each box represents the mean ± SE (n = 6). PC-3 cells were treated with various concentrations of cisplatin as indicated for 24 hours. Expressions of BTG2 were determined by immunoblotting assays (c) (Cropped gel) and RT-qPCR (d). (e) Luciferase activity of BTG2 promoter vectors-transfected PC-3 cells treated with various concentrations of cisplatin. Each value is the % in relation to the control (solvent-treated) group. Data are presented as the mean percentage ± SE (n = 6). (*P < 0.05, **P < 0.01).

We further evaluated the effect of cisplatin on cell proliferation of another prostate cancer cell, the p53-null PC-3 cells. Results of ^3^H-thymidine incorporation assay indicated cell proliferation of PC-3 cells decreased 42% and 54% when cells were treated with 40 and 80 μM, respectively, of cisplatin for 24 hours; whereas, cell proliferation decrease 34–87% after treatment with 10–80 μM of cisplatin for 48 hours (Figure 3a). Results from flow cytometric analysis revealed that 40 μM of cisplatin induced 20% increase in G1 phase cells together with a decrease in S phase cells of PC-3 cells after 24 hours incubation. High dosage of cisplatin (80 μM) induced PC-3 cell apoptosis indicated by the increased sub-G1 fraction cells (Figure 3b). The results of immunoblot assays indicated that cisplatin also induced BTG2 expression in PC-3 cells. Quantitative analysis revealed a 1.8-fold increase of BTG2 protein level after cisplatin 20 μM treatment (Figure 3c). RT-qPCR and transient gene expression assays also demonstrated that cisplatin upregulated BTG2 gene expression in a dose-dependent manner in PC-3 cells (Figure 3d, e). Of note, 80 μM cisplatin also induced less BTG2 expression as compared to 40 μM cisplatin in PC-3 cells, in line with the result in LNCaP cells.

**Figure 4:**
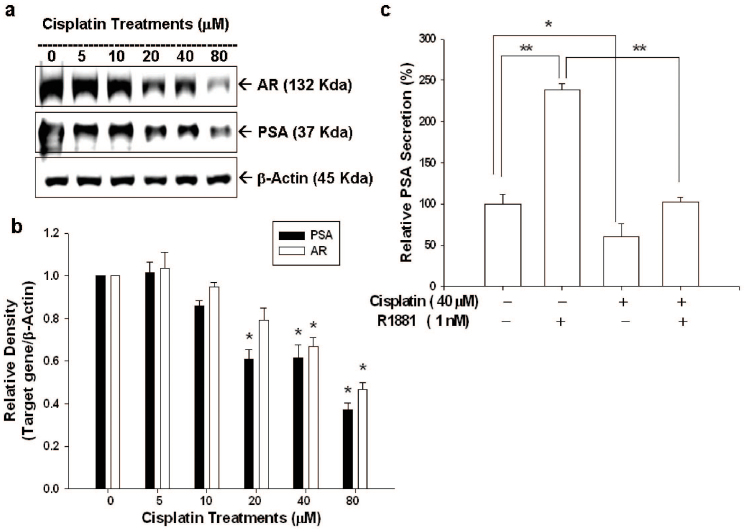
Cisplatin modulates androgen receptor and prostate-specific antigen expression in LNCaP cells. (a) LNCaP cells were treated with indicated concentrations cisplatin for 24 hours. Cells were lysed and expressions of androgen receptor (AR) ad prostate-specific antigen (PSA) were determined by immunoblotting assay. (Cropped gel) (b) Data of quantitative immunoblot analysis are expressed as relative density (mean ± SE; n = 3). (c) LNCaP cells were treated with 40 μM of cisplatin with or without R1881 (1 nM) for 24 hours. The conditional media were analyzed by ELISA for PSA concentrations. Each value is presented as % in relation to the control (solvent-treated) group. Data are shown as the mean percentage ± SE (n = 6). (*P < 0.05, **P < 0.01).

Results of immunoblot assays revealed that cisplatin blocked not only PSA but also AR expressions in a dose-dependent manner (Figure 4a). Quantitative analysis of immunoblot assays revealed that 80 μM of cisplatin decreased 63% and 54% of PSA and AR protein levels, respectively (Figure 4b). The ELISA assay indicated that cisplatin (40 μM) blocked the secretion of PSA in LNCaP cells. In addition, cisplatin also significantly blocked the stimulation of R1881 (1 nM) on PSA secretion (Figure 4c).

**Figure 5:**
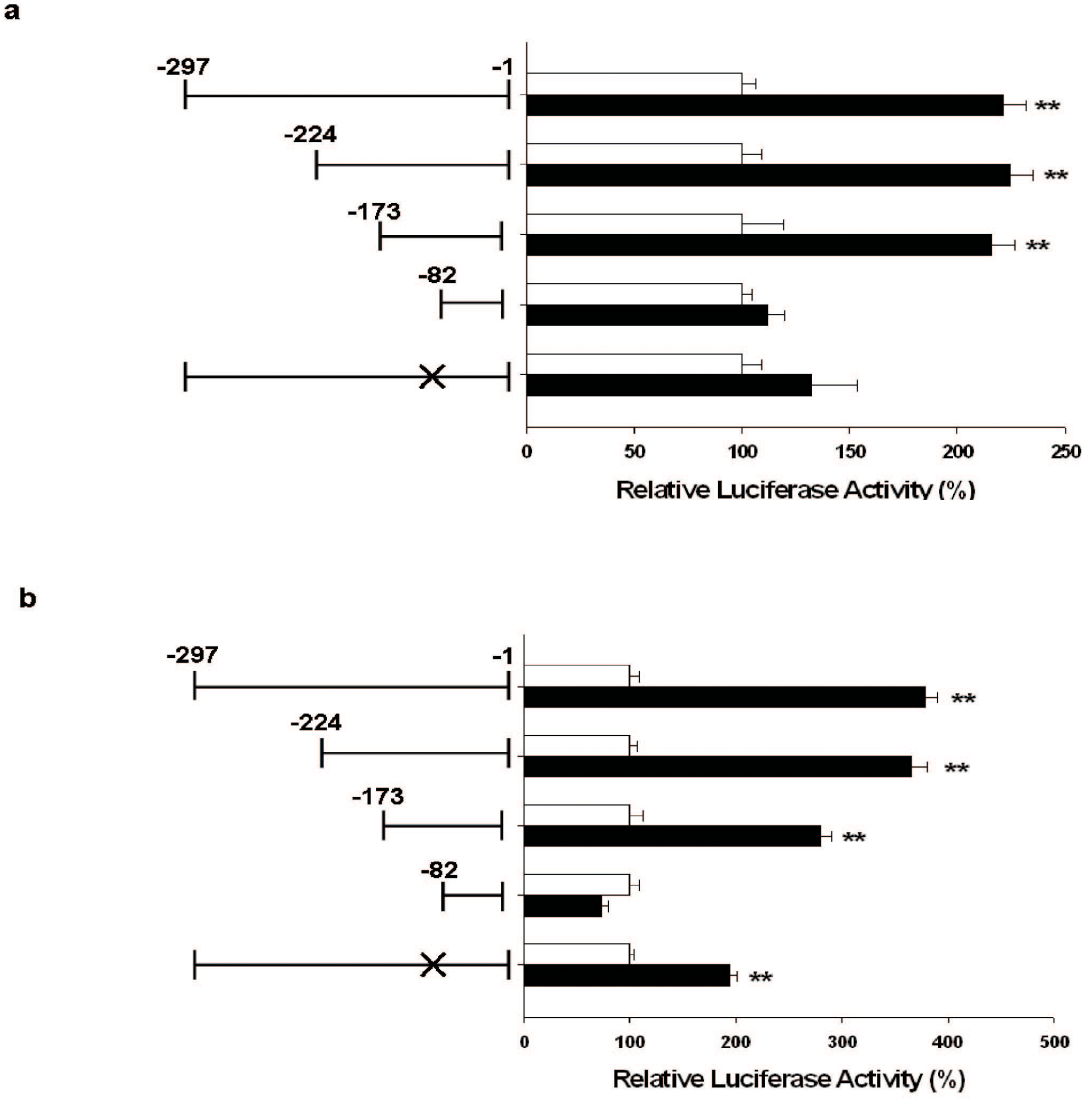
Cisplatin modulates BTG2 expression depending on DNA fragment 173 to −82 bp upstream of BTG2 gene p53-dependently or p53-independently in prostate carcinoma cells. Luciferase activity of nested deletion or mutation constructs BTG2 reporter vectors-transfected LNCaP (a) or PC-3 (b) cells after treatment of 40 μM of cispalatin (black bars) or control-solvent (white bars). X represented the mutation of p53 response element. Each value is the % in relation to the control solvent-treated group. Data are presented as the mean percentage ± SE (n = 6). (**P < 0.01).

Further transient BTG2 gene expression analyzed by reporter assays with 5′-deletion and site-mutation of p53 response element from GGGAAAGTCC to GGAGTCC within BTG2 promoter area showed that the effect of cisplatin on BTG2 gene expression not only depended on the p53 response element but also on the DNA fragment within −173 to −82 upstream of BTG2 translation initiation site in LNCaP cells (Figure 5a). Similar results were observed in PC-3 cells with the difference that site-mutation of p53 response element within BTG2 promoter area failed to abolish cisplatin-mediated BTG2 expressions (Figure 5b).

**Figure 6:**
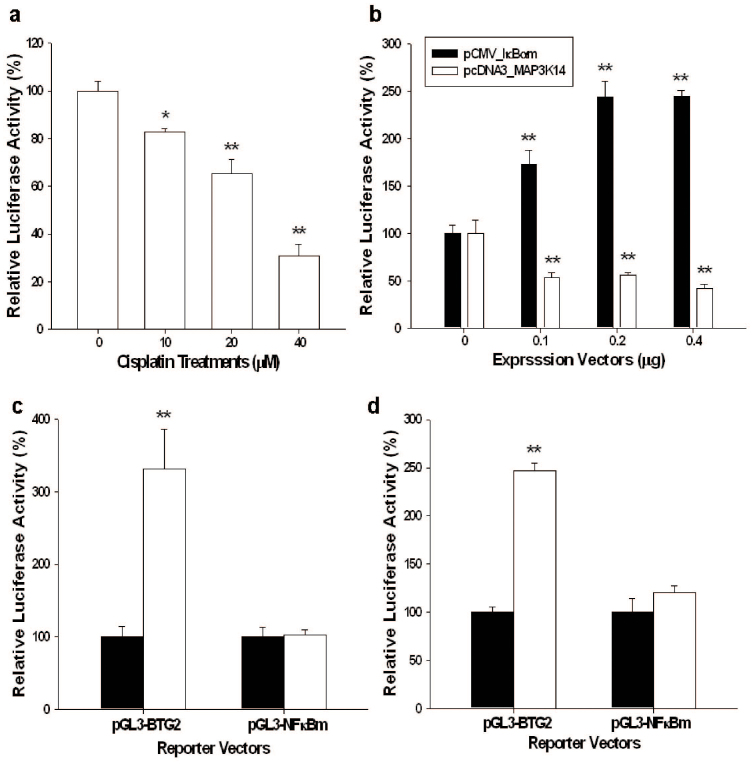
Cisplatin modulates BTG2 expression via the dwonregulation of NFκB activity in PC-3 cells. (a) Luciferase activity of NFκB reporter vectors-transfected PC-3 cells treated with indicated concentrations of cisplatin. (b) Luciferase activity of PC-3 cells cotrasfected with BTG2 reporter vectors along with IκB or MAP3K14 expression vectors. (c) Luciferase activity of wild-type BTG2 reporter vectors (pGL3-BTG2) - or BTG2 reporter vector with mutated NFκB response element (pGL3-NFκBm) -transfected PC-3 cells cotransfected with pcDNA3 (white bars) or IκB expression vector (black bars). (d). Luciferase activity of wild-type BTG2 reporter vectors (pGL3-BTG2)- or BTG2 reporter vector with mutated NFκB response element (pGL3-NFκBm)-transfected PC-3 cells treated with (black bars) or without (white bars) 20 μM cisplatin. Each value is the % in relation to the control group. Data are presented as the mean percentage ± SE (n = 6). (*P < 0.05, **P < 0.01).

Further transient gene expression assays with the NFκB reporter vector indicated that cisplatin treatments blocked the NFκB activity in a dose-dependent manner in PC-3 cells (Figure 6a).Transient overexpression IκBα induced BTG2 promoter activity while overexpression MAP3K14, one kind of NFκB-inducing kinase (NIK), downregulated BTG2 promoter activity (Figure 6b). A putative NFκB response element was found within −102 to −93 bp upstream of the translation starting point of the BTG2 gene. Transient overexpression IκBα (Figure 6c) or cisplatin (20 μM) (Figure 6d) treatment did not significantly enhance BTG2 promoter activity when the NFκB response element was mutated from GGAAAGTCC to GGAAAGGAA. Collectively, our result suggested that cisplatin modulated BTG2 expressions in PC-3 cells through the NFκB pathway.

## Discussion

Cisplatin, one of the potent antitumor agents, has been shown to have significant clinical activity against a variety of solid tumors and, thus, been listed as the standard regimens for some cancers treatment. The mechanisms by which cisplatin exerts cytotoxic mode of action mainly lie in the cisplatin interaction with DNA to form unique DNA adducts, which are usually intra strand crosslink adducts^15^. These DNA adducts would further modulate the expressions of several signal transduction pathways concerning the cell survival, such as p53, p73, ATR and MAPK. Among others, the activation of p53 is deemed as the major effect of cisplatin treatment to repress cancer cell growth^4^. However, cisplatin-based treatments often induces sever toxic side-effects which causes drug discontinuation and limit therapeutic efficacy^16^. Therefore, studies in recent reports tried to combine the targeting delivery or gene therapy to enhance therapeutic effect and safety of cisplatin on the prostate cancer in vivo^6^,^17^,^18^.

As shown in Figure 1a and 3a, cisplatin (0–80 μM) treatment for 24 or 48 hours repressed LNCaP and PC-3 cell proliferation dose-dependently. The lower concentrations of cisplatin (0–40 μM) treatment mainly induced cell cycle arrest at G0/G1 phase in both LNCaP and PC-3 cells as indicated by increased G1 phase cell percentage (Figure 1b and 3b). At higher dose (80 μM), cisplatin exerted apoptosis induction as demonstrated by increased sub-G1 phase cells and expression of c-PARP in prostate cancer cells (Figure 1b, 1c and 3b). Our results clearly indicated that cisplatin is a potent antitumor drug against prostate cancer *in vitro*.

p53, a well-known tumor suppressor gene, has been shown to have interaction with varied key transcription factors to involve in several important biological functions, such as DNA repair, apoptosis, cell cycle, apoptosis, senescence and angiogenesis. Thus, p53 plays an important role against cancer onset and progression, including prostate cancer^15^ and is the target to be induced for a number of chemotherapy drugs. Our previous studies have shown that topoisomerase inhibitors could induce p53 expression in LNCaP cells^19^,^20^. As shown in Figure 2a and 2b, cisplatin treatment (from 0–80 μM) induced p53 expression in LNCaP cell, a p53 wild type prostate cancer cell, as determined by western blot. Our results were in line with previous studies indicating that cisplatin treatments could induce p53 activity in prostate cancer^17^,^21^.

BTG2, originally known as one kind of the early growth response genes^22^, was first isolated from 3T3 fibroblasts cells^23^. So far, BTG2 has been demonstrated to involve in varied vital cellular functions, such as in cell cycle regulation, in which BTG2 has been found to be able to repress cell cycle progression at G1/S or G2/M phases and function as a pan cell cycle modulator in a cell- or tissue- specific manner^9^. Thus, BTG2 has been deemed as a tumor suppressor gene in a variety of cancers, including gastric, breast, bladder, and prostate cancer^13^,^24^,^25^,^26^,^27^,^28^. Our results indicated that pre-apoptosis dosage of cisplatin (≤40 μM) treatments upregulated BTG2 gene expression determining by immublotting, RT-qPCR, and transient gene expression assays in both LNCaP and PC-3 cells (Figure 2a, 2b, 2c, 2e, 3c, 3d, 3e). However, cisplatin treatment at apoptosis-inducing dosage (80 μM) decreased the BTG2 protein levels but the protein level was restored by cotreated with the proteasome inhbitior, MG132 (Figure 2d). These results are consistent with other reports that BTG2 protein is sensitive to the ubiquitin-proteasom system induced by genotoxic agents^14^,^29^.

Given that our previous study has shown that ectopic overexpression of BTG2 in PC-3 cells could inhibit cancer cell proliferation^13^, to further verify BTG2 role in cisplatin-induced inhibition on prostate cancer proliferation, we knocked down BTG2 expression by shRNA in LNCaP cells. The less proliferation inhibition noted in LNBTG2si cells by cisplatin clearly indicates that cisplatin-mediated LNCaP cell growth inhibition is partly dependent on BTG2 pathway (Figure 2f).

PSA, an androgen-upregulated protein, is well-used as a sensitive biomarker to detect the recurrence of prostate cancer^30^,^31^. PSA also has been shown to enhance prostate cancer cell invasion and facilitate refractory prostate cancer progression^32^,^33^. AR plays a vital role in androgen-dependent prostate cancer growth. Since p53 has been shown to be involved in negative regulation of AR activity^14^,^34^, we, therefore, also evaluated the AR expression and PSA expression in AR+ LNCaP cells after cisplatin treatment. As shown in Figure 4a and 4b, cisplatin blocked AR and PSA expressions of AR+ LNCaP cells in a dose dependent manner as determined by western blot and transient gene expression assays. In addition, cisplatin also blocked LNCaP cell PSA secretion and the R1881-mediated enhanced PSA secretion (Figure 4c). Taken together, our results are in agreement with previous study which also showed that cisplatin upregulated p53 expression and blocked AR and PSA expression in LNCaP cells^21^. Since the BTG2 has been regarded as the corepressor for AR in prostate carcinoma cells^35^, cisplatin may decrease the PSA expression p53-indirectly by upregulation of BTG2. Although we demonstrated cisplatin repressed AR and PSA expression in p53 wild-type LNCaP cells, however, we still can not rule out the possibility that the unidentified p53-independent pathway may also involve in the blocking effect of cisplatin on AR activity^21^.

Regarding BTG2 induction, two pathways have been demonstrated to involve in, which are p53 dependent^10^,^11^, or p53 independent PKC-δ pathway^12^. In this study, we demonstrated for the first time that cisplatin treatment induced BTG2 expression in both p53-wild LNCaP cells and p53-null PC-3 cells. Since previous reports indicated that a putative p53 response element (RRRCWWGYYYN_(0-13)_RRRCWWGYYY) is located within human BTG2 promoter area and site-mutation of p53 response element abolished p53-dependent BTG2 induction in prostate cancer cells^13^,^14^,^36^, to further investigate the mechanisms whereby cisplatin modulates BTG2 expression in p53-wild LNCaP cells and p53-null PC-3 cells, we therefore applied 5′-deltion method and site-directed mutation of p53 response element, combined with BTG2 reporter assay, to further study. As shown in Fig. 5a and 5b, cisplatin enhanced BTG2 gene expression dependent on the DNA fragment located within −173 to −82 upstream of BTG2 translation initiation site in both LNCaP and PC-3 cells. Mutation of the p53 response element from GGGCAGAGCCC to GGGCACC by site-directed mutagenesis abolished the stimulation of cisplatin on the BTG2 promoter activity in LNCaP cells with no influence in PC-3 cells. Based on our result, we thus concluded that cisplatin upregulates BTG2 expression in prostate cancer cells through both p53-dependent and -independent pathways.

The IKK/NF-κB signaling plays an important role as its aberrant expression is usually linked to human cancers^37^,^38^,^39^. Amid the latent state, NF-κBs are sequestered in the cytosol due to binding with IκB (the NFκB inhibitor). As stimulated, IκB is phosphorylated by activated IKK (IκB kinase) and degraded by proteasome, leading to the release of NF-κB and subsequent nuclear translocation to modulate gene expression^40^.

Since previous studies have suggested that a putative NFκB response element (5′-GGGRNWYYCC-3′) is located at 5′-flanking region of BTG2 gene^13^,^36^, we further evaluated whether cisplatin modulates BTG2 through the NFκB pathway in PC-3 cells. Our results of transient gene expression assays showed that cisplatin blocked NFκB activity in PC-3 cells (Figure 6a), in agreement with the previous report showing that cisplatin treatment perturbed the interaction between NFκB protein and NFκB response element^41^. As we blocked or induced NFκB pathway by transient overexpression of IκB or MAP3K14, BTG2 expressions in PC-3 cells were upregulated or downregulated as determined by BTG2 reporter assay, indicating NFκB is a deactivator of BTG2 expression in PC-3 cells (Figure 6b). These results are in agreement with previous study showing curcumin upregulated BTG2 expression by blocking the NFκB activity in U937 cells, a human leukemic monocyte lymphoma cell line^42^ although other early study indicated that NFκB is the activator of BTG2 expression in T47D cells, a human ductal breast epithelial tumor cell line^43^. Further transient gene expression assays showed cisplatin treatment lost the activation on BTG2 promoter activity when the NFκB response element was mutated from GGAAAGTCC to GGAAAGGAA in the BTG2 reporter vector, confirming that cisplatin upregulated BTG2 expression through the NFκB pathway in PC-3 cells.

Based on our results, we concluded that cisplatin is a potent drug to repress prostate cancer growth in vitro through induction of cell cycle arrest at G0/G1 and apoptosis. Cisplatin blocks AR and PSA expressions while increased p53 and BTG2 expressions. Cisplatin-mediated growth inhibition in p53 wild-type LNCaP and p53 null PC3 cells is partly dependent on induction of BTG2 expression, which is modulated in either p53-dependent (LNCaP cells) or NF-κB (PC-3 cells), p53-independent, pathway.

## Methods

### Materials, cell lines, and cell culture

LNCaP and PC-3 cell lines were obtained and maintained as described previously^30^. Cisplatin and MG132 were purchased from Sigma (St. Louis, MO, USA), and. Methyltrienolone (R1881) was purchased from NEN life Science (Boston, MA, USA). The RPMI-1640 culture media were purchased from Life Technologies (Rockville, MD, USA). Fetal calf serum (FCS) was purchased from the HyClone (Logan, Utah, USA).

### Cell proliferation assay

Cell proliferation in response to cisplatin was measured using a ^3^H-thymidine incorporation assay as described previously^21^ or measured using a CyQUANT cell proliferation assay kit as described by manufacturer (Invitrogen, Carlsbad, CA, USA).

### Flow cytometry

Cells were serum starved for 24 hours and then cultured in RPMI 1640 medium with 10% FCS and with or without different concentrations of drugs for another 24 hours. Cell cycle analysis was performed using the FACS-Calibur cytometer and CellQuestPro software (BD Biosciences, San Jose, CA, USA); the data were analyzed using ModFit LT Mac 3.0 software as described previously^20^.

### Immunoblot assay

Equal quantities of cell extract were loaded onto a 12% sodium dodecyl sulfate polyacrylamide (SDS) gel and analyzed by the electrochemiluminescent detection system. The blotting membranes were probed with 1:500 polyclonal BTG2 antiserum^12^, 1:500 diluted polyclonal PSA antiserum (A0562, DakoCytomation, Glostrup, Denmark), 1:200 diluted human androgen receptor antiserum (N-20; Santa Cruz Biotechnology, Santa Cruz, CA, USA), 1:500 diluted human p53 antiserum (DO-1; Santa Cruz Biotechnology), or 1:3000 diluted β-actin antiserum (I-19, Santa Cruz Biotechnology). The intensity of different bands were recorded and analyzed by GeneTools of ChemiGenius (Syngene, Cambridge, UK).

### PSA enzyme-linked immunosorbent assay

The PSA levels in the conditional media were measured by PSA enzyme linked immunosorbent assay (ELISA) and was adjusted by the concentration of protein in the whole cell extract as described previously^31^.

### BTG2 knockdown

LNCaP cells were transduced with BTG2 small hairpin RNA lentiviral particles (Sc-43645-V; Santa Cruz Biotechnology) as described by the manufacturer. Two days after transduction, the cells (LN-BTG2si) were selected with 5 μg/ml puromycin dihydrochloride. The mock-transfected LNCaP cells (LN-COLsi) were transduced with control small hairpin RNA lentiviral particles (Sc-10808-V, Santa Cruz Biotechnology) and were clonally selected in the same manner as the BTG2-knockdown cells.

### Reverse transcriptase- real-time polymerase chain reaction

Total RNA was isolated using Trizol reagent, and cDNA was synthesized and real-time polymerase chain reactions (qPCR) were performed using an ABI StepOne Plus Real-Time PCR system (Applied Biosystems, Foster City, CA, USA) as described previously^20^. FAM dye-labeled TaqMan MGB probes and PCR primers for BTG2 (Hs00198887_ml) were purchased from Applied Biosystems. Glyceraldehyde 3-phosphate dehydrogenase (GAPDH; Hs99999905_m1) was used as the internal positive control.

### Expression vector constructs

The pCMV-IκBαm expression vector which contains serine to alanine mutations at residues 32 and 36 of IκBα was purchased from Clontech (Moutain View, GA, USA). The MAP3K14 expression vector was constructed by cloning the MAP3K14 cDNA (MGC:45335; Invitrogen) after digestion with *Eco RI* and *Xho I* into the pcDNA3 expression vector (Invitrogen).

### Report vector constructs and reporter assay

The NFκB reporter vector was purchased from Clontech. The reporter vectors containing 5′-flanking region -1 to −297, −1 to −244, −1 to −173, and −1 to −82 of the human BTG2 gene was cloned as modified from previously study^12^. The reporter vectors were constructed by mutation of p53 response element from GGGCAGAGCCC to GGGCACC and by mutation of NFκB response element from GGAAAGTCC to GGAAAGGAA of BTG2 gene. Cells were seeded onto 24-well plates at 1 × 10^4^ cells/well 1 day prior to transfection. The cells were transiently transfected with 1 μg/well of reporter vectors as indicated and 0.5 μg/well of β-galactosidase expression vector (pCMVSPORTβgal; Life Technologies) as described previously^30^. The activities of luciferase and β-galactosidase were assayed as specified by the manufacturer instructions (Promega Bioscience, San Luis Obispo, CA, USA).

### Statistical analysis

Results are expressed as the mean ± S.E. of at least three independent replication of each experiment. Statistical significance was determined by one-way ANOVA and Student's *t* test using the SigmaStat program for Window version 2.03 (SPSS Inc, Chicago, IL, USA).

## Author Contributions

K.-C.C. and K.-H.T. wrote the manuscript and designed this experiment, L.-C.C., C.-N.Y., T.-H.F., W.-T.C., P.-L.C. and H.-Y.C.J. helped conduct the experiment, H.-H.J. was in charge of the whole experiment conduction and paper writing.
